# Diagnostic stewardship perceptions and practices among microbiology laboratories: a national survey from the American Society for Microbiology

**DOI:** 10.1128/jcm.00239-26

**Published:** 2026-06-22

**Authors:** Rosemary C. She, Paige Larkin, J. Scott Parrott, Ryan Tom, Rebekah E. Dumm, Logan Patterson, Laura M. Filkins

**Affiliations:** 1 Department of Pathology, City of Hope 378541 , Duarte, California, USA; 2 American Society for Microbiology 11003, Washington, DC, USA; 3 Department of Interdisciplinary Studies, Rutgers School of Health Professions, Rutgers University 67206 , Newark, New Jersey, USA; 4 Department of Clinical Laboratory & Medical Imaging Sciences, Rutgers School of Health Professions, Rutgers University 67206 , Newark, New Jersey, USA; 5 Department of Pathology and Immunology, Washington University School of Medicine 12275 , St. Louis, Missouri, USA; 6 Department of Pathology & Laboratory Medicine, Medical University of South Carolina 2345, Charleston, South Carolina, USA; 7 Department of Pathology, University of Texas Southwestern Medical Center 12334, Dallas, Texas, USA; Maine Medical Center Department of Medicine, Portland, Maine, USA

**Keywords:** information technology in diagnostics, laboratory utilization, infectious disease diagnostics, clinical microbiology

## Abstract

Diagnostic stewardship (DxS) is a key activity for providing effective and efficient testing strategies for patient care. Complex test menus and result interpretation, high costs, limited reimbursements, and other factors necessitate DxS practices in clinical microbiology. We conducted a national survey of clinical microbiologists on their perceptions and practice of DxS in infectious diseases (ID) diagnostics. The 119 respondents (83 clinical directors, 33 operational leaders, and three trainees) indicated that DxS was practiced at their institution “some” (50.4%) or “most” (23.5%) of the time. Most respondents (>95%) had infection prevention and control and antimicrobial stewardship committees at their institutions. Academic medical center laboratories (AMCs) more often reported having microbiology consultation service, DxS/laboratory utilization committee, or official laboratory formulary than community hospital laboratories (*P* ≤ 0.001). Respondents identified ID providers (79.0%) and clinical laboratory directors (74.8%) as the most common participants in their DxS activities. Leveraging informational technology (IT) was considered the most effective strategy (78.2%), and antimicrobial stewardship and/or ID collaboration (52.1%) was the most valuable resource for supporting DxS. The biggest challenge was time and personnel resources (52.1%). Respondents highlighted molecular testing, including syndromic panels (22.7%) and sequencing-based assays (21.0%), as a top priority for DxS efforts, followed by culture-based testing (20.2%). AMC respondents more likely considered sequencing assays as highest priority than other respondents (*P* < 0.001). These data provide a valuable baseline for understanding the state of DxS in clinical microbiology laboratories. Key challenges include personnel resources and opportunities include engaging with ID providers and utilization of IT tools for DxS initiatives.

**IMPORTANCE:**

At a time when microbiology test menus are expanding with costly molecular diagnostics while workforce shortages and reimbursement constraints persist, we conducted a national survey-based study of clinical microbiology professionals to capture their perceptions and practice of diagnostic stewardship. Results from 119 clinical microbiologists identified several common challenges as well as key differences between respondents from different institutional types as well as between respondents fulfilling different professional roles. The survey additionally identified diagnostic stewardship interventions regarded as most effective, factors most important in prioritization of stewardship efforts, and breadth of roles participating in diagnostic stewardship for microbiology tests. These unique observations into the state of diagnostic stewardship in clinical microbiology laboratories help establish a baseline for future research and guideline development in infectious disease testing.

## INTRODUCTION

The concept of diagnostic stewardship (DxS) identifies a longstanding, critical aspect in the practice of laboratory medicine and its subdisciplines, including clinical microbiology. DxS in laboratory testing refers to ensuring selection of the clinically appropriate test, use of the appropriate analytical method, accurate reporting of applicable results, and correct interpretation and understanding of test results to provide optimal patient care. Clinical microbiology laboratories have long carried out DxS practices as required by regulatory agencies, accrediting bodies, training programs, and fiscal responsibility (1). Facing operational challenges such as workforce shortages in microbiology technologists (2), increase in number of high-cost molecular test options (3), and limited test reimbursement despite increasing healthcare costs (4), combined with increasingly complex test menu offerings that require careful test selection and result interpretation to optimize patient management, continued DxS efforts are a requisite for accurate, judicious, and efficient use of microbiology laboratory tests and resources.

The present need to enhance DxS in clinical microbiology is further underscored by increasing clinical concern for antimicrobial resistant pathogens. It is now widely recognized that interdisciplinary efforts are necessary to institute effective antimicrobial stewardship (AMS) (5). AMS has broadened beyond its initial focus on antibiotic prescription to encompass directly related processes, including stewardship of clinical microbiology testing, to further support the goals of AMS (5, 6). Recommended DxS strategies for key microbiology tests are designed to improve pretest probability for pathogen detection and thereby better align test usage and results with need for antimicrobial therapy (7, 8).

The American Society for Microbiology (ASM) has actively engaged in review and creation of guidelines in DxS as a resource for laboratories (1, 9, 10). As members of the Laboratory Practices Subcommittee (LPS), we identified a gap in understanding baseline DxS practices and perceptions across clinical microbiology laboratories. As advances in technology outpace the development of clinical outcome studies, reimbursement rules, and cost-benefit analyses, gauging the challenges in DxS faced by clinical microbiology laboratories in today’s environment is crucial to creating meaningful guidelines and policies. Survey-based studies have been performed to better understand DxS practices for AMS (8, 11, 12), for specific infectious diseases tests (13–15), and in clinical laboratories broadly (12, 16). Such studies have yet to assess the state of DxS specifically in clinical microbiology laboratories to understand baseline perceptions and practices. Resources and test utilization patterns differ by institution, and it is important to understand which challenges are shared or are unique between laboratories.

Herein, we describe the results from a national survey of clinical microbiology professionals on their perceptions and practices of DxS at their own institutions as an initiative of the ASM LPS. The intent of the study was to gain a perspective on the current state of DxS, identify trends in the perception of DxS in practice, and understand areas of need for implementation and success of DxS in clinical microbiology laboratories.

## MATERIALS AND METHODS

### Survey design and administration

A 12-question survey was designed to assess the state of DxS activities and programs within the clinical microbiology laboratory community (Supplemental Data: Survey Questions). Three questions gathered information on survey respondent demographics, including institution, professional role within the laboratory, and type of microbiology laboratory in which they worked. Three questions aimed to capture information on the current DxS practices at their institution, and six questions captured data on their perceptions of DxS characteristics that were effective, as well as gaps in DxS practices. Response choices were constructed based on established practices and the peer-reviewed literature on DxS (1, 9, 17, 18). The survey was created using the web-based Microsoft Forms, and the survey link was distributed by email to two ASM listservs, one for clinical microbiology directors (i.e., ClinMicroNet) and one for clinical microbiologists at any level (i.e., DivC), in April 2024 and again in June 2024. Only two questions required responses (role in the laboratory and type of microbiology laboratory, Questions 2 and 3); thus, a variable number of responses were possible for the other questions. One question (Q11: top current test priority for DxS) requested exclusively free-text responses. Those responses were interpreted and classified within broader categories by the study personnel. For some institutions, more than one participant responded to the survey. Multiple responses from a single institution were analyzed as independent responses (i.e., no data were excluded based on the site). The rationale for not excluding data was two-fold. First, not all participants responded to the question about their institution; thus, there was no way to ensure complete removal of multiple responses. Second, the primary aim of this study was to assess perceptions, and we determined that individual respondents from the same institution could have different perceptions worth capturing. Survey responses were voluntary, and no incentives were provided.

### Statistical analysis

Descriptive data (n and %) were tabulated for each question’s responses, and additional stratified analyses were performed by the role of the survey respondent (clinical director role vs operational leader role) as well as by the type of laboratory (academic medical center laboratory [AMC], hospital system reference laboratory, or community hospital laboratory) Table 1. Trainee roles were excluded from stratified analyses by role in the laboratory due to their small number (*n* = 3) and stage in practice compared to other respondents. Commercial reference laboratories, governmental laboratories, and responses that indicated more than one laboratory type served were excluded from stratified analyses by laboratory type due to small numbers. To protect against family-wise error (inflated Type 1 error), a two-step approach was used for the analyses of the association between roles and lab types. First, due to the fact that we were examining a configuration of different decision choices for each question, each of which measured categorically, an omnibus test (nonparametric permutational analysis of variance [19]) was used to determine whether, across question options, there was a significant association between respondent choices and both role and lab type. Assuming omnibus significance (*P* < 0.05), the results were decomposed to identify the significance of specific characteristics or features differing by role or lab type. Finally, a chi-square analysis was performed on significant associations to evaluate patterns in question responses by role and laboratory type. *P*-values < 0.05 were considered statistically significant. The R statistical package was used for all analyses, and the vegan package was used for permutational analysis of variance.

**TABLE 1:** Demographic data of survey respondents

Role in the microbiology laboratory	N
Clinical director roles	83
Medical/Technical Director	80
Medical/Technical Director/Administrative Director	1
CLIA Director	2
Operational laboratory leader roles	33
Lead/Senior Technologist	13
Supervisor	11
Manager	6
Administrative Director	1
Lead/Senior/Supervisor	1
Supervisor/Manager	1
Trainees	3
Post-Doctoral Fellow	3
Total number of responses	119

Type of microbiology laboratory	
Academic medical center laboratory	68
Community hospital laboratory	28
Hospital system reference laboratory	18
Other/multiple laboratories	5
Both academic medical center and commercial reference lab	1
Both academic medical center and reference lab for the system	1
Commercial reference laboratory	2
Government medical center laboratory	1
Total number of responses	119

## RESULTS

### Demographic data

The survey was completed by 119 unique individuals from at least 82 different institutions; 24 respondents did not provide institution information. Institutions were based in 28 U.S. states and six different countries (Table S1). By professional role, respondents represented clinical directors (*n* = 83, 69.7%), operational leaders (*n* = 33, 27.7%), and trainees (*n* = 3, 2.5%) of clinical microbiology laboratories. Respondents served laboratories at AMC (*n* = 68, 57.1%), community hospitals (*n* = 28, 23.5%), hospital system reference laboratories (*n* = 18, 15.1%), more than one laboratory type (*n* = 2, 1.7%), or others (*n* = 3, 2.5%). Table 1 provides further details. Clinical director respondents were more frequently associated with AMC laboratories (*P* < 0.001), and operational leader respondents were more frequently associated with community hospital laboratories (*P* < 0.001) (Table 2).

**TABLE 2:** Distribution of respondent roles in the microbiology laboratory and the laboratory setting with which they were affiliated

	Type of microbiology laboratory				
Role in microbiology lab	Academic medical center laboratory	Community hospital laboratory	Hospital system reference laboratory	Other/ multiple laboratories	Total
Clinical director	58 (48.7%)	7 (5.9%)	13 (10.9%)	5 (4.2%)	83 (69.7%)
Operational leader	7 (5.9%)	21 (17.6%)	5 (4.2%)	0 (0.0%)	33 (27.7%)
Trainee	3 (2.5%)	0 (0.0%)	0 (0.0%)	0 (0.0%)	3 (2.5%)
Total	68 (57.1%)	28 (23.5%)	18 (15.1%)	5 (4.2%)	119 (100%)

The survey response rate is estimated to be 7.6%–13.8% but could not be precisely calculated because the number of overlapping members between DivC and ClinMicroNet Listservs and the number of inactive Listserv members are unknown. Estimates were based on the minimum and maximum potential overlapping membership and assuming no inactive members, using the reported membership numbers for ClinMicroNet (860) and DivC (699) as displayed in the ASM Listservs portal around the time of survey distribution.

### DxS practices of survey respondents

When asked about existing committees or system processes related to DxS at their institution, most indicated there were infection prevention and control (116/119, 97.5%) and antimicrobial stewardship program (115/119, 96.6%) committees (Table 3 ; Table S2). The least commonly reported committees or processes were an official laboratory formulary (16.8%, 20/119) or “other” (2.5%, 3/119). An average of 4.5 (median 4, SD 1.91, range 0–8) committees/systems were selected out of the eight options presented (excluding the “other” free-text option). When the responses were analyzed against the laboratory setting of the respondent, three committees or processes were significantly associated with it: AMC laboratory respondents were more likely than those of community hospital laboratories to report having a microbiology consultation service (58.8% vs 10.7%, *P* < 0.001), DxS/lab utilization committee (55.9% vs 17.9%, *P* < 0.001), or official laboratory test formulary (25.0% vs 0%, *P* = 0.001).

**TABLE 3:** Survey responses on DxS practices by laboratory practice setting^*a*^

Question	Academic medical center laboratory (*n* = 68)	Community hospital laboratory (*n* = 28)	Hospital reference laboratory (*n* = 18)	Other/multiple laboratories (*n* = 5)	Total (*n* = 119)
Institution Committees/Processes: Which of the following committees or system processes exist at your institution? (select all that apply)^*b*^					
IPC committee	67 (98.5%)	28 (100.0%)	17 (94.4%)	4 (80.0%)	116 (97.5%)
AMS committee	66 (97.1%)	28 (100.0%)	17 (94.4%)	4 (80.0%)	115 (96.6%)
Restricted microbiology test approval	45 (66.2%)	12 (42.9%)	8 (44.4%)	3 (60.0%)	68 (57.1%)
IT new order review	43 (63.2%)	11 (39.3%)	10 (55.6%)	3 (60.0%)	67 (56.3%)
Microbiology consultation	40 (58.8%)	3 (10.7%)	6 (33.3%)	3 (60.0%)	52 (43.7%)
DxS/lab utilization committee	38 (55.9%)	5 (17.9%)	5 (27.8%)	1 (20.0%)	49 (41.2%)
Referral testing utilization review	29 (42.6%)	10 (35.7%)	7 (38.9%)	3 (60.0%)	49 (41.2%)
Official lab formulary	17 (25.0%)	0 (0.0%)	1 (5.6%)	2 (40.0%)	20 (16.8%)
Other^*c*^	0 (0%)	1 (3.6%)	1 (5.6%)	1 (20.0%)	3 (2.5%)
No options were selected	0 (0%)	0 (0%)	1 (5.6%)	0 (0%)	1 (0.8%)

Roles participating in diagnostic stewardship: Which of the following roles actively participate in diagnostic stewardship for.infectious disease tests at your institution? (select all that apply)^a^					
ID providers and trainees	52 (76.5%)	25 (89.3%)	13 (72.2%)	4 (80.0%)	94 (79.0%)
Medical/Scientific Director	56 (82.4%)	15 (53.6%)	14 (77.8%)	4 (80.0%)	89 (74.8%)
AMS pharmacists and trainees	44 (64.7%)	22 (78.6%)	10 (55.6%)	3 (60.0%)	79 (66.4%)
Manager/supervisors	30 (44.1%)	20 (71.4%)	9 (50.0%)	4 (80.0%)	63 (52.9%)
Ordering providers	20 (29.4%)	9 (32.1%)	3 (16.7%)	4 (80.0%)	36 (30.3%)
Pathology/microbiology trainees	26 (38.2%)	4 (14.3%)	3 (16.7%)	2 (40.0%)	35 (29.4%)
Operational Director	20 (29.4%)	8 (28.6%)	5 (27.8%)	1 (20.0%)	34 (28.6%)
Laboratory technologists	12 (17.6%)	7 (25.0%)	3 (16.7%)	1 (20.0%)	23 (19.3%)
Other pathologists	15 (22.1%)	3 (10.7%)	1 (5.6%)	1 (20.0%)	20 (16.8%)
None/not sure	4 (5.9%)	4 (14.3%)	2 (11.1%)	0 (0%)	10 (8.4%)
C-suite	5 (7.4%)	2 (7.1%)	2 (11.1%)	0 (0%)	9 (7.6%)
Other trainees	3 (4.4%)	1 (3.6%)	1 (5.6%)	0 (0%)	5 (4.2%)
No options were selected	1 (1.5%)	0 (0%)	0 (0%)	0 (0%)	1 (0.8%)

^*a*^ AMS, antimicrobial stewardship; DxS, diagnostic stewardship; IPC, infection prevention and control; ID, infectious diseases; IT, information technology.

^*b*^ One participant did not select any responses for these questions and was counted as not having or being involved in any of the listed committees/system processes.

^*c*^ Summarized “Other” free text responses (n = 3): ID/Pharmacy/Microbiology meeting; Value Analysis Team; The Diagnostic Stewardship Committee has become inactive.

Respondents were then asked if they were directly involved in any of those committees or processes. Five (4.2%) participants did not indicate participation in any of the committees or processes. The most common committees or processes in which respondents indicated direct participation were the antimicrobial stewardship program committee (103/119, 86.6%) and Infection Prevention and Control Committee (96/119, 80.7%) (Table 4). A low proportion of respondents (14/119, 11.8%) indicated participation in an official laboratory test formulary, which corresponded with the overall low reporting of the existence of such a process at their institution. However, 70% (14/20) of respondents who indicated the existence of an official laboratory test formulary reported directly participating in the activity. Respondents who reported committees or systems related to DxS at their institution (excluding responses of “other”) directly participated in 44.8%–96.2% depending on the committee or system (Table S3). There were some inconsistencies in the responses, wherein a few respondents indicated that they directly participated in a committee or process but did not indicate its existence at their institution, which may be due to a difference in the wording of the two questions specifying the existence of a “system process” versus participation in a “process.” The greatest discrepancy was observed from respondents who indicated participating in a microbiology clinical call consultation system without indicating the existence of a microbiology clinical call system process at their institution (*n* = 5). An average of 3.6 (median 3, SD 1.97, range 0–8) committees/systems were selected out of the eight options presented (excluding the “other” free-text option). When the responses were analyzed against the laboratory role of the respondent, clinical directors were significantly more likely than operational leaders to participate in each of the committees or processes listed (all *P* < 0.02), except for “information technology new order review committee or process.”

**TABLE 4:** Survey responses to DxS practices and perceptions by the role of the respondent^*a*^

Question	Clinical Director (*n* = 83)	Operational Leader (*n* = 33)	Total (*n* = 119)^*b*^
Respondent Committee Participation: In which of the following committees or processes are you directly involved? (select all that apply)^*c*^			
AMS committee	79 (95.2%)	22 (66.7%)	103 (86.6%)
IPC committee	75 (90.4%)	19 (57.6%)	96 (80.7%)
Restricted microbiology test approval	55 (66.3%)	3 (9.1%)	60 (50.4%)
Microbiology consultation	46 (55.4%)	6 (18.2%)	55 (46.2%)
DxS/lab utilization committee	37 (44.6%)	3 (9.1%)	40 (33.6%)
IT new order review	25 (30.1%)	7 (21.2%)	32 (26.9%)
Referral testing utilization review	26 (31.3%)	3 (9.1%)	29 (24.4%)
Official lab formulary	14 (16.9%)	0 (0%)	14 (11.8%)
Other^*d*^	1 (1.2%)	0 (0%)	1 (0.8%)
No options were selected	0 (0%)	5 (15.2%)	5 (4.2%)

Most Effective DxS Practices: Which of the following activities do you consider to be most effective for diagnostic stewardship? (select up to 4)			
IT test order rules	70 (84.3%)	22 (66.7%)	93 (78.2%)
Upholding specimen requirements	51 (61.4%)	20 (60.6%)	74 (62.2%)
AST selective/cascade reporting	42 (50.6%)	23 (69.7%)	66 (55.5%)
Microbiology consultation	36 (43.4%)	10 (30.3%)	49 (41.2%)
Result interpretative comments	20 (24.1%)	16 (45.5%)	35 (29.4%)
Manual process for test approvals	28 (33.7%)	3 (9.1%)	33 (27.7%)
Test catalog	13 (15.7%)	10 (30.3%)	24 (20.2%)
Test utilization feedback	16 (19.3%)	7 (21.2%)	23 (19.3%)
Memos and bulletins	12 (14.5%)	10 (30.3%)	22 (18.5%)
Targeted education	14 (16.9%)	3 (9.1%)	17 (14.3%)
Reimbursement denial feedback	3 (3.6%)	3 (9.1%)	6 (5.0%)
No response	2 (2.4%)	1 (3.0%)	3 (2.5%)

Biggest Challenges: What are the biggest challenges you experience in practicing diagnostic stewardship in your position? Select at most two options.			
Time and personnel resources	42 (50.6%)	18 (54.5%)	62 (52.1%)
IT resources	40 (48.2%)	9 (27.3%)	50 (42.0%)
Provider support	33 (39.8%)	3 (9.1%)	38 (31.9%)
Institutional support	21 (25.3%)	9 (27.3%)	31 (26.1%)
Financial resources	5 (6.0%)	12 (36.4%)	17 (14.3%)
Relationship with other departments	7 (8.4%)	3 (9.1%)	10 (8.4%)
Lab staff education	4 (4.8%)	4 (12.1%)	8 (6.7%)
Other^*e*^	2 (2.4%)	4 (12.1%)	6 (5.0%)
Client support	2 (2.4%)	0 (0%)	2 (1.7%)
No response	1 (1.2%)	1 (3.0%)	2 (1.7%)

DxS Performance: How consistently do you think your institution adequately performs diagnostic stewardship for the infectious disease tests?			
Not at all	5 (6.0%)	0 (0%)	5 (4.2%)
Very little	17 (20.5%)	5 (15.2%)	23 (19.3%)
Some of the time	48 (57.8%)	12 (36.4%)	60 (50.4%)
Most of the time	12 (14.5%)	14 (42.4%)	28 (23.5%)
Always	1 (1.2%)	2 (6.1%)	3 (2.5%)
No response	0 (0%)	0 (0%)	0 (0%)

^*a*^ AST, antimicrobial susceptibility testing; DxS, diagnostic stewardship; IT, information technology.

^*b*^ Trainee (n = 3) responses are not shown due to low numbers but are included in the tabulations of the total (n = 119).

^*c*^ Five participants (operational leadership roles) did not select any responses and were counted as negative responses to all potential selections.

^*d*^ “Other” free text response (n = 1): ID/Pharmacy/Microbiology meeting.

^*e*^ Summarized “Other” free text responses (n = 6): lack of visibility/opportunity for involvement; lack of collaboration between the administration and laboratory; lack of exposure to DxS; culture within the healthcare system; staff shortages; lack of laboratory medical director support.

When prompted with a definition of DxS (“activities intended to improve clinical laboratory test ordering, performance, and/or reporting to optimize patient treatment and outcomes”), respondents were asked to select all the staff roles that actively participate in DxS for infectious disease tests at their institution. Most respondents indicated that ID providers (including fellows) (94/119, 79.0%) or medical/scientific directors (89/119, 74.8%) were actively involved (Table 3; Table S2), whereas <10% indicated that C-suite and trainees outside of pathology and ID actively participated. AMC laboratory respondents were more likely than community hospital laboratory respondents to indicate that medical/scientific directors (82.4% vs 53.6%, *P* = 0.008) and microbiology trainees (38.2% vs 14.3%, *P* = 0.037) participated in DxS (Table 3).

### Perception of DxS practices

Participants were asked to select up to four out of a set of 11 DxS activities in ID testing that they considered to be most effective (Table 4). The most selected response was “developing information technology rules for test orders (restrictions, reflexes, BPAs)” (93/119, 78.2%). This was followed by “upholding pre-analytic specimen requirements” (74/119, 62.2%), “selective and cascade reporting of antimicrobial susceptibility results” (66/119, 55.5%), and “microbiology call consultation services” (49/119, 41.2%). Less than one-third of survey respondents selected the other activities listed. Of note, compared to operational leaders, clinical directors were significantly more likely to select “manual process for denying or approving test requests” (33.7% vs 9.1%, *P* = 0.005) or “developing information technology rules for test orders” (84.3% vs 66.7%, *P* = 0.022) as the most effective tools. But when responses were analyzed against the laboratory type of the respondents, no difference was observed between AMC and community hospital laboratory types in selecting “developing IT rules for test orders” (79.4% vs 82.1%, respectively; Table S4). AMC laboratory respondents were less likely than community hospital or hospital system reference laboratories to indicate “selective and cascade reporting of antimicrobial susceptibility results” as the most effective strategy (44.1% vs 71.4% and 77.8%, respectively; *P* = 0.01).

Next, participants were asked to rank the importance of the following six resources for the success of DxS: AMS and/or ID team support, IT support (e.g., test builds, best practice advisories, and hard stops), clinical providers' support, laboratory operations support, analytic reports for developing or monitoring interventions, and financial support from their institution. The most common selection as most important was AMS and/or ID team support (62/119, 52.1%), followed by IT support (e.g., test builds, best practice advisories, and hard stops) (selected as most important by 18/119, 15.1% and as second-most important by 44/119, 37.0%; Tabe S5). Respondents ranked financial support from the institution as the least important resource (65/119, 54.6%). Overall, operational leaders ranked financial support significantly higher than clinical directors (*P* = 0.004), whereas clinical directors ranked IT support significantly higher than operational leaders (*P* < 0.001). Compared to respondents from community hospital laboratories and hospital reference laboratories, respondents from AMC laboratories ranked analytical reports for interventions (*P* = 0.006) and IT support (*P* = 0.01) as more important and financial support as less important (*P* = 0.04).

More varied responses were received when participants were asked to select the biggest two challenges to DxS from a list of eight options (Table 4). Slightly more than half believed that time and personnel resources were the biggest challenges (62/119, 52.1%). Support from IT resources (50/119, 42.0%) or clinical providers (38/119, 31.9%) was also a common selection. Less than 30% selected institutional support (31/119, 26.1%), financial resources (17/119, 14.3%), laboratory relationship with other departments (10/119, 8.4%), laboratory staff education (8/119, 6.7%), or client support (2/119, 1.7%). Based on respondents’ role type, clinical directors were significantly more likely to indicate clinical provider support as one of the biggest challenges to practicing DxS (*P* = 0.002), whereas operational leaders were more likely to see financial resources as an obstacle (*P* < 0.001).

When respondents were asked to rank six factors that could affect test prioritization for DxS (test turnaround time, test cost, risk of misinterpretation or misuse of results, test performance [negative and positive predictive values], personnel resource constraints, and frequency of mis-ordering or overutilization), “risk of misinterpretation or misuse of results” was ranked in the top two in importance by 68.6% (81/118) of respondents, followed by “frequency of mis-ordering or overutilization” (65/119, 54.6%). The two drivers most frequently ranked as lowest two in importance were “personnel resource constraints” (77/119, 64.7%) and “test turnaround time” (66/119, 55.5%) (Table S5). When considering respondents’ role types, clinical directors ranked the risk of misinterpretation or misuse of results (*P* < 0.001) and frequency of mis-ordering or overutilization (*P* < 0.001) significantly higher than operational leaders. Conversely, compared to clinical directors, operational leaders ranked test turnaround time (*P* < 0.001) and test cost (*P* = 0.04) more highly as drivers for DxS prioritization.

Finally, when asked how consistently they thought their institution was adequately performing DxS for ID tests, most respondents selected “some of the time” (60/119, 50.4%), “most of the time” (28/119, 23.5%), or “very little” (23/119, 19.3%). Only three (2.5%) responded “always,” and five (4.2%) responded “not at all” (Table 4). When responses were assessed in relation to the respondents’ role or laboratory type, operational leadership roles indicated higher levels of consistency in their perception of how adequately their institution performs DxS compared to clinical director roles (*P* = 0.003). Community hospital laboratory respondents also indicated higher levels of consistency in DxS practice in their perspective compared to AMC laboratory and hospital reference laboratory-type respondents (*P* = 0.02; Table S4).

### Current DxS needs

When asked to name one test (free-text) to prioritize for DxS, 108 (90.8%) participants provided a response (Table 5). The most frequently cited tests belonged to the molecular microbiology testing category (55/119, 46.2%), including syndromic molecular panels (*n* = 27), sequencing (*n* = 25) (including broad-range sequencing, next-generation sequencing [NGS], cell-free DNA metagenomic NGS [cfDNA mNGS]), and PCR tests (*n* = 3). Respiratory syndromic panel (*n* = 12) and cfDNA mNGS (*n* = 15) were the most commonly specified molecular assays. Culture-based tests were named by 24 (20.2%) respondents, including urine culture (*n* = 11), blood culture (*n* = 8), mycobacterial culture (*n* = 4), and wound culture (*n* = 1). Seven (5.9%) responses listed susceptibility testing (cascade reporting, improving turnaround time, or general AMS efforts), and three (2.5%) responses each were received for *C. difficile* and ova and parasite testing. Various serology tests were named by eight (6.7%) participants. The remaining responses were related to general systems or test categories.

**TABLE 5:** Response to question, “If only able to focus on one test, which test would you prioritize for your efforts towards diagnostic stewardship today?” according to the respondent role^*a*^

Response: test category	Clinical director (*n* = 83)	Operational leader (*n* = 33)	Total (*n* = 119)^*b*^
Syndromic panel	22 (26.5%)	5 (15.2%)	27 (22.7%)
Sequencing/mNGS	23 (27.7%)	1 (3.0%)	25 (21.0%)
Culture	18 (21.7%)	5 (15.2%)	24 (20.2%)
Serology	5 (6.0%)	2 (6.1%)	8 (6.7%)
AST	1 (1.2%)	6 (18.2%)	7 (5.9%)
*C. difficile*	2 (2.4%)	1 (3.0%)	3 (2.5%)
Ova and parasite exam	1 (1.2%)	2 (6.1%)	3 (2.5%)
PCR	0 (0%)	3 (9.1%)	3 (2.5%)
Other^*c*^	5 (6.0%)	3 (9.1%)	8 (6.7%)
No response	6 (7.2%)	5 (15.2%)	11 (9.2%)

^*a*^ AST, antimicrobial susceptibility testing; mNGS, metagenomic next-generation sequencing.

^*b*^ Trainee (n = 3) responses are not shown due to low numbers but are included in the tabulations of the total (n = 119).

^*c*^ Includes non-microbiology tests (C-reactive protein, n = 1) and nonspecific tests or categories: stool testing (n = 2), high-cost molecular (n = 1), high-volume tests (n = 1), LIS support (n = 1), any send out (n = 1), and MALDI (n = 1).

When analyzing the category of the selected tests according to the role of the respondents, clinical directors were more likely to list molecular microbiology sequencing tests (*P* = 0.004) as a priority, and operational leaders were more likely to list antimicrobial susceptibility testing (*P* = 0.002) as a priority for DxS (Table 5). Similarly, when analyzing responses according to the laboratory type of the respondents, AMC laboratory respondents were more likely to indicate molecular microbiology sequencing tests (*P* = 0.004) as a priority compared to community hospital laboratory or hospital reference laboratory respondents (Table S6).

## DISCUSSION

We present a unique cross-sectional analysis of general DxS perceptions and practices of clinical microbiology laboratory professionals with 119 survey respondents from across the United States and a small number from five other countries. Use and availability of DxS tools and activities varied greatly by institution type as well as according to the laboratory role of the respondent, but we were able to discern some overall trends and key patterns in responses. We believe that data from this survey can be used to understand where guidelines may focus efforts and help develop multidisciplinary partnerships on the subject.

Overall, most respondents communicated that DxS was carried out at least some of the time at their institution, but very few perceived that DxS was applied consistently all the time. Time and personnel resources were the most cited challenge to successful DxS implementation across all laboratory types and respondent roles, likely reflecting the constancy and investment required for effective DxS along with longstanding workforce shortages in clinical laboratories (20). Other major challenges included IT resources and clinical provider support. There were some notable areas of differences in the perception of DxS between operational leadership and clinical directorship roles. Operational leaders perceived financial resources to be a significant barrier to DxS (37.5%), in contrast to clinical directors (6%). Test cost was also a larger driver of DxS prioritization according to operational leaders compared to clinical directors, whereas clinical directors more often indicated prioritizing tests based on the risk of misinterpretation or test misutilization. From this, we recognize the importance of not only overlapping priorities but also the different roles and contributions of laboratory team members in DxS activities. Inclusion of both operational leaders and clinical directors as laboratory stakeholders would broaden the perspectives of the DxS team.

Use and availability of DxS tools and activities varied greatly between survey respondents, except for those mandated by regulations in the United States, namely, AMS and infection prevention and control (IPC). Existence of other DxS committees or systems was less frequent, though overall, multiple (median of 4) tended to exist at the institutions surveyed, and respondents tended to participate in more than one (median of 3). These findings are encouraging on the state of DxS development in today’s laboratories, and others have shown that use of multiple DxS interventions is associated with greater success (17, 18).

Our sub-analysis demonstrated that academic medical centers had more DxS committees/systems than institutions associated with the other laboratory types, and clinical directors participated more frequently in DxS-related systems and activities than operational leaders. These findings are somewhat expected considering AMCs are more likely to have a dedicated clinical director in microbiology, and many DxS activities, such as clinical consultations and test approvals, fall under the scope of clinical practice. Our surveyed professionals indicated that ID providers and pharmacists were frequently involved in DxS of ID tests, although we do not know based on the survey questions asked if this was due solely to their role in AMS and IPC committees versus additional activities. Other survey studies have found AMS and DxS initiatives to be more difficult in hospitals without multidisciplinary teams or expertise in ID (21). These observations serve to emphasize that the optimal DxS program profile for a given institution depends on many factors, including the existence of a clinical microbiologist and other roles at the institution. Regardless, test utilization initiatives often originate from laboratory members of DxS committees (12), and laboratory support is required for accurate data collection, monitoring, and analysis (1, 5).

With the increased use of costly molecular sequencing technologies in ID testing, a widely held assumption is that DxS of DNA sequencing-based tests would be a top contemporary priority for microbiology laboratories (22). New molecular tests such as mNGS lack historical context and evidence-based guidelines for how to use them and interpret results. Such guidelines for broad-spectrum, pathogen-agnostic tests require time to develop and are most impactful when derived from decades of experience and clinical studies. While this survey confirmed that molecular tests were the most cited DxS priority, they were more often cited by clinical directors and AMC laboratory respondents than operational leaders or by respondents associated with other laboratory types. This finding is complemented by the fact that conventional tests, such as culture, antimicrobial susceptibility testing, and serologies, were named as top priorities for DxS by one-third of respondents, across categories of the role and laboratory type. These results capture the continued issues with inappropriate culture ordering, especially routine blood and urine cultures, which remain the subject of present-day reviews in ID DxS (5, 7, 23, 24). Our survey data underscore the breadth of ID testing that is relevant to DxS and the importance of DxS for all microbiology laboratories as a quality assurance activity.

While other DxS survey-based studies related to microbiology testing have focused mainly on AMS, specific tests or syndromes, or clinician perspectives (13, 25, 26), they have also provided insights into focused topics related to clinical microbiology laboratories. The Society for Healthcare Epidemiology of America (SHEA) Research Network, for example, has examined DxS to support optimal use of respiratory PCR panels as well as AMS in the setting of respiratory PCR panel testing (14, 15). In these studies, only slightly over half of sites reported having some form of DxS interventions for test ordering. Interventions considered to be effective included use of electronic order sets, test restrictions, and communication or education with providers. Another survey of clinical laboratories involved in DxS similarly found IT support in addition to access to data to be valuable resources for successful projects (12). In further support of implementing IT decision support tools in DxS, one DxS survey found its application in febrile pediatric patients to be highly accepted by pediatric intensivists as it supports appropriate microbiologic work-up and standardizes practices across different providers in defined circumstances (13). Our survey of microbiology respondents similarly found IT support to be selected by most (78.2%) as an effective DxS tool. Information technology, including artificial intelligence, will play an increasing role in healthcare systems as tools increase in sophistication for decision support. Particularly in DxS, IT support can both enforce institutional policies and help educate providers on appropriate testing at the time of ordering, without requiring laboratory intervention. Notably, routine laboratory practices such as upholding specimen requirements (62.2% of respondents), and optimizing result reporting, such as employing AST selective and cascade reporting (55.5%), were selected among the most effective methods, underscoring the important role of routine quality assurance practices in DxS.

As an ASM membership-based survey, our data are derived from clinical microbiology professionals largely in the U.S. DxS practices differ greatly between different countries and healthcare systems, and this study may not be applicable to other locations. A global survey (11) on AMS and DxS that included under-resourced areas found that access to diagnostic interventions for improving AMS is limited in many areas, yet most providers perceived that when instituted, DxS practices led to improved patient outcomes and resulted in metrics supporting AMS. Other studies have demonstrated that under-resourced areas face difficulties in accessing resources, and underutilization of routine microbiology tests is a major challenge (27, 28), in contrast to overutilization and cost control being noted as common drivers of DxS in our survey responses.

Limitations in this survey-based study include that because the survey was administered on different dates and email distribution lists, duplicates may have occurred especially as data were anonymous. However, we noted that among respondents from the same institution as another respondent (*n* = 16), survey responses were not identical and therefore were not considered duplicates. As a survey-based study, questions and response choices were subject to the potential for variable interpretation. We found a few minor inconsistencies in the responses between related questions for a given respondent, as noted in the Results section. A small number of respondents did not answer all survey questions, and for questions 4 and 5 (yes/no choices), we did not provide a choice of “none of the above” to confirm the lack of response as a “negative” rather than a non-response. Representation heavily favored clinical directorship roles in AMC or reference laboratories and favored operational leadership roles in community hospital labs. Therefore, the respondent’s role and the type of laboratory in which they serve were not entirely independent variables. Additionally, there were almost no responses by executive laboratory leadership (i.e., a single respondent identifying as an administrative director without a clinical role). As survey participation was voluntary, willingness to participate in the survey may introduce response bias or predilection for a respondents’ preconceived notions of DxS. This survey also only reached ASM members, specifically those who participate in one or both listservs used to distribute the survey. Finally, the survey did not assess the respondent level of experience or years at the present institution or the number of doctoral microbiologists, breadth of test menu, or patient population, as pertains to the institution of the respondent.

In summary, this survey captured the opinions and state of DxS activities in clinical microbiology laboratories. The biggest perceived challenge was time and personnel resources, while opportunities included engaging with ID providers and utilization of IT tools to support DxS initiatives. Further comprehensive studies on DxS in clinical microbiology are needed, especially those that include greater representation from community hospital laboratories and laboratories without dedicated clinical microbiologists. Such studies would help illuminate the broader landscape of DxS across diverse fields. Other areas that warrant surveillance include DxS practices in the direct-to-consumer testing setting, the specific needs of different patient populations (e.g., quaternary, tertiary, secondary, and primary care facilities), and differences between outpatient and inpatient DxS practices. DxS topics not covered by our survey, such as value-based payment methods, reimbursement incentives, perceived outcomes of DxS, and role of guidelines and other expert resources, also merit further investigation.

## SUPPLEMENTAL MATERIAL

Supplemental Materials jcm.00239-26-s0001.docx Survey questionnaire and Tables S1 to S6.

ASM does not own the copyrights to Supplemental Material that may be linked to, or accessed through, an article. The authors have granted ASM a non-exclusive, world-wide license to publish the Supplemental Material files. Please contact the corresponding author directly for reuse.
