# Supplementary material for: Diagnostic stewardship perceptions and practices among microbiology laboratories: a national survey from the American Society for Microbiology
Source: J Clin Microbiol. 2026 Jun 22;64(8):e00239-26. doi: 10.1128/jcm.00239-26 (PMC13464345; doi:10.1128/jcm.00239-26)
Supplement: Supplementary file 1 [file jcm.00239-26-s0001.docx]

**Supplemental Data**

**Survey Questions**

1. Name of your institution: (response not required, this will be used only for analysis purposes if multiple participants reply from a single institution)^a^
2. Please indicate your role in the clinical microbiology laboratory.^b^

- Lead/Senior Technologist
- Supervisor
- Manager
- Administrative Director
- Post-Doctoral Fellow
- ACGME Resident/Fellow
- Medical/Technical Director
- CLIA Director

3. Please indicate the type of microbiology lab in which you primarily work in:^b^

- Academic medical center laboratory
- Community hospital laboratory
- Hospital system reference laboratory
- Commercial reference laboratory
- Public health laboratory
- Other^a^

4. Which of the following committees or system processes exist at your institution? (select all that apply)

- Antimicrobial stewardship program committee
- Infection prevention and control committee
- Diagnostic stewardship committee/Laboratory test utilization committee
- Referral testing utilization review committee or process (e.g. test-specific volume review, assessment for bringing in tests)
- Information technology new order review committee or process (e.g. IT change control, oversight of inclusion of a lab test on a departmental order set)
- Microbiology clinical call consultation system
- Restricted microbiology test order approval process
- Official laboratory formulary
- Other^a^

5. In which of the following committees or processes are you directly involved? (select all that apply)

- Antimicrobial stewardship program committee
- Infection Prevention and control committee
- Diagnostic stewardship committee/Laboratory test utilization committee
- Referral testing utilization review committee or process (e.g. test-specific volume review, assessment for bringing in tests)
- Information technology new order review committee or process (e.g. IT change control, oversight of inclusion of a lab test on a departmental order set)
- Microbiology clinical call consultation system
- Restricted microbiology test order approval process
- Official laboratory formulary
- Other^a^

For the rest of this survey, please apply the following definition when considering your responses. Diagnostic stewardship may be defined as activities intended to improve clinical laboratory test…

6. Which of the following roles actively participate in diagnostic stewardship for infectious disease tests at your institution? (select all that apply)

- Medical or Scientific Director
- Ordering providers
- Infectious disease providers, including ID fellows
- Pathology or Microbiology trainees (residents, fellows)
- Other trainees outside of pathology and infectious diseases
- Antimicrobial stewardship pharmacists or trainees
- Other pathologists
- Operational (or administrative) laboratory director
- Microbiology laboratory manager and supervisors
- Microbiology laboratory technologists
- None to my knowledge/not sure
- C-suite
- Other^a^

7. Which of the following activities do you consider to be most effective for diagnostic stewardship? (select up to 4)

- Microbiology call consultation services
- Manual process for denying or approving test requests
- Upholding pre-analytic specimen requirements
- Issuing memos/bulletins with laboratory testing changes or guidance
- Providing clinical department or subspecialty-specific test utilization feedback
- Providing clinical department or subspecialty-specific reimbursement denials feedback
- Maintaining a detailed test catalog
- Developing information technology rules for test orders (restrictions, reflexes, BPAs)
- Developing result interpretative comments
- Selective and cascade reporting of antimicrobial susceptibility results
- Targeted topic in-service provider or nursing education (e.g., ordering, collection techniques)
- Other^a^

8. Rank the following resources from most to least important when developing diagnostic stewardship for infectious disease tests. (Drag and drop with the most important at the top.)

- Antimicrobial stewardship and/or infectious disease team support
- Clinical providers' support
- Information technology (IT) support (e.g. test builds, best practice advisories, hard stops)
- Analytic reports for developing or monitoring interventions
- Laboratory operations support
- Financial support from institution

9. What are the biggest challenges you experience in practicing diagnostic stewardship in your position? Select at most 2 options.

- Institutional support
- Clinical provider support
- Financial resources
- Time and personnel resources
- Information technology resources
- Lab staff education
- Client support (for reference or system labs)
- Laboratory relationship with other departments
- Other^a^

10. Rank the aspects that drive your prioritization of which test(s) to steward (drag and drop with most important at the top).

- Test turnaround time
- Test cost
- Risk of misinterpretation or misuse of results
- Test performance (NPV, PPV)
- Personnel resource constraints
- Frequency of mis-ordering or overutilization

11. Current needs: If only able to focus on one test, which test would you prioritize for your efforts towards diagnostic stewardship today?^a^
12. How consistently do you think your institution adequately performs diagnostic stewardship for the infectious disease tests?^c^

- Always
- Most of the time
- Some of the time
- Very little
- Not at all

^a^Free-text response

^b^Multiple responses allowed (no limit)

^c^Only one response allowed

**Supplemental Table 1.** Geographic distribution of survey respondents (n=94 responses).

| **State (US)** | **Number of Respondents** |
| --- | --- |
| Alabama | 1 |
| Arizona | 1 |
| California | 7 |
| Colorado | 3 |
| Connecticut | 3 |
| Florida | 4 |
| Georgia | 4 |
| Illinois | 4 |
| Iowa | 1 |
| Kansas | 3 |
| Maryland | 7 |
| Michigan | 3 |
| Minnesota | 1 |
| Missouri | 5 |
| New Hampshire | 1 |
| New Jersey | 2 |
| New Mexico | 1 |
| New York | 6 |
| North Carolina | 7 |
| Ohio | 4 |
| Oregon | 1 |
| Pennsylvania | 4 |
| South Dakota | 1 |
| Texas | 5 |
| Vermont | 1 |
| Virginia | 3 |
| Washington | 2 |
| Washington, D.C. | 1 |
| Wisconsin | 2 |
| **Country (non-US)** |  |
| Canada | 2 |
| Ghana | 1 |
| Greece | 1 |
| Lebanon | 1 |
| Nepal | 1 |
| **Total** | **94** |

**Supplemental Table 2.** Survey responses to DxS practices by role of the respondent.

| **Question** | **Clinical Director (n=83)** | **Operational Leader (n=33)** | **Total (n= 119)^a^** |
| --- | --- | --- | --- |
| **Institution Committees/Processes:** Which of the following committees or system processes exist at your institution? (select all that apply)^b^ | | | |
| IPC committee | 82 (98.8%) | 31 (93.9%) | 116 (97.5%) |
| AMS committee | 82 (98.8%) | 31 (93.9%) | 115 (96.6%) |
| Restricted microbiology test approval | 56 (67.5%) | 9 (27.3%) | 68 (57.1%) |
| IT new order review | 51 (61.4%) | 16 (48.5%) | 67 (56.3%) |
| Microbiology consultation | 44 (53.0%) | 5 (15.2%) | 52 (43.7%) |
| DxS/Lab utilization committee | 44 (53.0%) | 5 (15.2%) | 49 (41.2%) |
| Referral testing utilization review | 37 (44.6%) | 11 (33.3%) | 49 (41.2%) |
| Official lab formulary | 20 (24.1%) | 0 (0%) | 20 (16.8%) |
| Other^c^ | 3 (3.6%) | 0 (0%) | 3 (2.5%) |
| No options were selected | 0 (0%) | 1 (3.0%) | 1 (0.8%) |
| **Roles Participating in Diagnostic Stewardship:** Which of the following roles actively participate in diagnostic stewardship for infectious disease tests at your institution? (select all that apply)^b^ | | | |
| ID providers & trainees | 65 (78.3%) | 26 (78.8%) | 94 (79.0%) |
| Medical/Scientific Director | 72 (86.7%) | 15 (45.5%) | 89 (74.8%) |
| AMS pharmacists & trainees | 55 (66.3%) | 22 (66.7%) | 79 (66.4%) |
| Manager /supervisors | 39 (47.0%) | 23 (69.7%) | 63 (52.9%) |
| Ordering Providers | 27 (32.5%) | 9 (27.3%) | 36 (30.3%) |
| Pathology/Microbiology trainees | 26 (31.3%) | 7 (21.2%) | 35 (29.4%) |
| Operational Director | 25 (30.1%) | 8 (24.2%) | 34 (28.6%) |
| Laboratory technologists | 14 (16.9%) | 8 (24.2%) | 23 (19.3%) |
| Other pathologists | 19 (22.9%) | 1 (3.0%) | 20 (16.8%) |
| None/Not sure | 5 (6.0%) | 15.2% | 10 (8.4%) |
| C-suite | 8 (9.6%) | 1 (3.0%) | 9 (7.6%) |
| Other trainees | 4 (4.8%) | 1 (3.0%) | 5 (4.2%) |
| No options were selected | 1 (1.2%) | 0 (0%) | 1 (0.8%) |

Abbreviations: AMS, antimicrobial stewardship; DxS, diagnostic stewardship; IPC, infection prevention and control; ID, infectious diseases; IT, information technology

^a^ Trainee (n=3) responses are not shown due to low numbers but are included in the tabulations of the total (n=119).

^b^ One participant (operational leadership role) did not select any responses for these questions and was counted as not having or being involved in any of the listed committees/system processes.

^c^ Summarized “Other” free text responses (n=3): ID/Pharmacy/Microbiology meeting; Value Analysis Team; DxS committee has become inactive.

**Supplemental Table 3:** Survey Question Response Pattern Comparing Questions 4 and 5.

|  | Committee/ System Process Exists | Committee/ System Process Exists and Respondent Participates | Committee/ System Process Exists but Respondent Does Not Participate | Committee/ System Process Does Not Exist but Respondent Participates | Participation of Those Reporting the System Exists |
| --- | --- | --- | --- | --- | --- |
| AMS | 115 | 103 | 12 | 0 | 103/115 (89.6%) |
| Diagnostic stewardship | 49 | 38 | 11 | 2 | 38/49 (77.6%) |
| IPC | 116 | 96 | 20 | 0 | 96/116 (82.8%) |
| IT new order review | 67 | 30 | 37 | 2 | 30/67 (44.8%) |
| Microbiology consultation | 52 | 50 | 2 | 5 | 50/52 (96.2%) |
| Official formulary | 20 | 14 | 6 | 0 | 14/20 (70.0%) |
| Other | 3 | 1 | 2 | 0 | 1/3 (33.3%) |
| Referral testing | 49 | 26 | 23 | 3 | 26/49 (53.1%) |
| Restricted microbiology | 68 | 57 | 11 | 3 | 57/68 (83.8%) |

Abbreviations: AMS, antimicrobial stewardship; IPC, infection prevention and control; IT, information technology

**Supplemental Table 4.** Survey responses to DxS perceptions by type of laboratory of the respondent.

| **Question** | **Academic Medical Center Laboratory (n=68)** | **Community Hospital Laboratory (n=28)** | **Hospital Reference Laboratory (n=18)** | **Other/ Multiple Laboratories (n=5)** | | **Total (n=119)** | |
| --- | --- | --- | --- | --- | --- | --- | --- |
| **Respondent Committee Participation:** In which of the following committees or processes are you directly involved? (select all that apply)^a^ | | | | | | | |
| AMS committee | 61 (89.7%) | 23 (82.1%) | 15 (83.3%) | 4 (80.0%) | | 103 (86.6%) | |
| IPC committee | 58 (85.3%) | 20 (71.4%) | 15 (83.3%) | 3 (60.0%) | | 96 (80.7%) | |
| Restricted microbiology test approval | 42 (61.8%) | 8 (28.6%) | 8 (44.4%) | 2 (40.0%) | | 60 (50.4%) | |
| Microbiology consultation | 40 (58.8%) | 4 (14.3%) | 8 (44.4%) | 3 (60.0%) | | 55 (46.2%) | |
| DxS/Lab utilization committee | 31 (45.6%) | 4 (14.3%) | 5 (27.8%) | 0 (0%) | | 40 (33.6%) | |
| IT new order review | 25 (36.8%) | 5 (17.9%) | 1 (5.6%) | 1 (20.0%) | | 32 (26.9%) | |
| Referral testing utilization review | 18 (26.5%) | 6 (21.4%) | 4 (22.2%) | 1 (20.0%) | | 29 (24.4%) | |
| Official lab formulary | 12 (17.6%) | 0 (0.0%) | 1 (5.6%) | 1 (20.0%) | | 14 (11.8%) | |
| Other^d^ | 0 (0%) | 0 (0%) | 1 (5.6%) | 0 (0%) | | 1 (0.8%) | |
| No options were selected | 1 (1.5%) | 2 (7.1%) | 2 (11.1%) | 0 (0%) | | 5 (4.2%) | |
| **Most Effective DxS Practices:** Which of the following activities do you consider to be most effective for diagnostic stewardship? (select up to 4) | | | | | | |  |
| IT test order rules | 54 (79.4%) | 23 (82.1%) | 13 (72.2%) | 3 (60.0%) | 93 (78.2%) | |  |
| Upholding specimen requirements | 40 (58.8%) | 20 (71.4%) | 9 (50.0%) | 5 (100.0%) | 74 (62.2%) | |  |
| AST selective/cascade reporting | 30 (44.1%) | 20 (71.4%) | 14 (77.8%) | 2 (40.0%) | 66 (55.5%) | |  |
| Microbiology consultation | 32 (47.1%) | 6 (21.4%) | 7 (38.9%) | 4 (80.0%) | 49 (41.2%) | |  |
| Result interpretative comments | 23 (33.8%) | 10 (35.7%) | 2 (11.1%) | 0 (0%) | 35 (29.4%) | |  |
| Manual process for test approvals | 22 (32.4%) | 4 (14.3%) | 7 (38.9%) | 0 (0%) | 33 (27.7%) | |  |
| Test catalog | 13 (19.1%) | 5 (17.9%) | 5 (27.8%) | 1 (20.0%) | 24 (20.2%) | |  |
| Test utilization feedback | 12 (17.6%) | 7 (25.0%) | 3 (16.7%) | 1 (20.0%) | 23 (19.3%) | |  |
| Memos & bulletins | 10 (14.7%) | 7 (25.0%) | 4 (22.2%) | 1 (20.0%) | 22 (18.5%) | |  |
| Targeted education | 11 (16.2%) | 3 (10.7%) | 1 (5.6%) | 2 (40.0%) | 17 (14.3%) | |  |
| Reimbursement denials feedback | 3 (4.4%) | 2 (7.1%) | 1 (5.6%) | 0 (0%) | 6 (5.0%) | |  |
| No response | 2 (2.9%) | 0 (0%) | 1 (5.6%) | 0 (0%) | 3 (2.5%) | |  |
| **Biggest Challenges:** What are the biggest challenges you experience in practicing diagnostic stewardship in your position? Select at most 2 options. | | | | | | |  |
| Time & personnel resources | 39 (57.4%) | 13 (46.4%) | 10 (55.6%) | 0 (0%) | 62 (52.1%) | |  |
| IT resources | 34 (50.0%) | 8 (28.6%) | 5 (27.8%) | 3 (60.0%) | 50 (42.0%) | |  |
| Provider support | 21 (30.9%) | 6 (21.4%) | 9 (50.0%) | 2 (40.0%) | 38 (31.9%) | |  |
| Institutional support | 15 (22.1%) | 6 (21.4%) | 9 (50.0%) | 1 (20.0%) | 31 (26.1%) | |  |
| Financial resources | 5 (7.4%) | 10 (35.7%) | 2 (11.1%) | 0 (0%) | 17 (14.3%) | |  |
| Relationship with other departments | 6 (8.8%) | 3 (10.7%) | 0 (0%) | 1 (20.0%) | 10 (8.4%) | |  |
| Lab staff education | 5 (7.4%) | 3 (10.7%) | 0 (0%) | 0 (0%) | 8 (6.7%) | |  |
| Other^b^ | 3 (4.4%) | 3 (10.7%) | 0 (0%) | 0 (0%) | 6 (5.0%) | |  |
| Client support | 1 (1.5%) | 0 (0%) | 0 (0%) | 1 (20.0%) | 2 (1.7%) | |  |
| No response | 1 (1.5%) | 1 (3.6%) | 0 (0%) | 0 (0%) | 2 (1.7%) | |  |
| **DxS Performance:** How consistently do you think your institution adequately performs diagnostic stewardship for the infectious disease tests? | | | | | | |  |
| Not at all | 4 (5.9%) | 0 (0%) | 1 (5.6%) | 0 (0.0%) | 5 (4.2%) | |  |
| Very little | 12 (17.6%) | 4 (14.3%) | 5 (27.8%) | 2 (40.0%) | 23 (19.3%) | |  |
| Some of the time | 36 (52.9%) | 11 (39.3%) | 10 (55.6%) | 3 (60.0%) | 60 (50.4%) | |  |
| Most of the time | 14 (20.6%) | 12 (42.9%) | 2 (11.1%) | 0 (0.0%) | 28 (23.5%) | |  |
| Always | 2 (2.9%) | 1 (3.6%) | 0 (0.0%) | 0 (0.0%) | 3 (2.5%) | |  |
| No response | 0 (0%) | 0 (0%) | 0 (0%) | 0 (0%) | 0 (0%) | |  |

Abbreviations: AST, antimicrobial susceptibility testing; DxS, diagnostic stewardship; IT, information technology

^a^ Five participants (operational leadership roles) did not select any responses and were counted as negative responses to all potential selections.

^b^ Summarized “Other” free text responses (n=6): lack of visibility/opportunity for involvement; lack of collaboration between administration and laboratory; lack of exposure to DxS; culture within the healthcare system; staff shortages; lack of laboratory medical director support.

**Supplemental Table 5.** Responses to ranking questions 8 and 10.

| **Rank the following resources from most to least important when developing diagnostic stewardship for infectious disease tests (1=most important, 6=least important)** | | | | | | |
| --- | --- | --- | --- | --- | --- | --- |
| **Rank** | **1** | **2** | **3** | **4** | **5** | **6** |
| AMS and/or ID team support | 62 (52.1%) | 22 (18.5%) | 15 (12.6%) | 11(9.2%) | 5 (4.2%) | 1 (0.8%) |
| IT support | 18 (15.1%) | 44 (37.0%) | 24 (20.2%) | 16 (13.5%) | 12 (10.1%) | 2 (2.5%) |
| Clinical providers support | 12 (10.1%) | 24 (20.2%) | 26 (21.9%) | 16 (13.5%) | 23 (19.3%) | 15 (12.6%) |
| Lab operations support | 10 (8.4%) | 9 (7.6%) | 23 (19.3%) | 32 (26.9%) | 30 (25.2%) | 12 (10.1%) |
| Analytic reports | 2 (1.7%) | 10 (8.4%) | 21 (17.7%) | 32 (26.9%) | 30 (25.2%) | 21 (17.7%) |
| Financial support | 12 (10.1%) | 7 (5.9%) | 7 (5.9%) | 9 (7.6%) | 16 (13.5%) | 65 (54.6%) |
| No Response | 3 (2.5%) | 3 (2.5%) | 3 (2.5%) | 3 (2.5%) | 3 (2.5%) | 3 (2.5%) |
| **Rank the aspects that drive your prioritization of which test(s) to steward (1=most important, 6=least important)** | | | | | | |
| **Rank** | **1** | **2** | **3** | **4** | **5** | **6** |
| Risk of misinterpretation or misuse of results | 43 (36.1%) | 38 (31.9%) | 16 (13.4%) | 10 (8.4%) | 8 (6.7%) | 3 (2.5%) |
| Frequency of mis-ordering or overutilization | 35 (29.4%) | 30 (25.2%) | 25 (21.0%) | 9 (7.6%) | 8 (6.7%) | 11 (9.2%) |
| Test cost | 26 (21.8%) | 19 (16.0%) | 24 (10.2%) | 27 (22.7%) | 9 (7.6%) | 13 (10.9%) |
| Test performance (NPV, PPV) | 9 (7.6%) | 14 (11.8%) | 29 (24.4%) | 25 (21.0%) | 35 (29.4%) | 6 (5.0%) |
| Test turnaround time | 1 (0.8%) | 13 (10.9%) | 12 (10.1%) | 26 21.8%) | 27 (22.7%) | 39 (32.8%) |
| Personnel resource constraints | 4 (3.4%) | 4 (3.4%) | 12 (10.1%) | 21 (17.6%) | 31 (26.1%) | 46 (38.7%) |
| No response | 1 (0.8%) | 1 (0.8%) | 1 (0.8%) | 1 (0.8%) | 1 (0.8%) | 1 (0.8%) |

Abbreviations: AMS, antimicrobial stewardship; ID, infectious diseases; IT, information technology; NPV, negative predictive value; PPV, positive predictive value

**Supplemental Table 6.** Response to question, “If only able to focus on one test, which test would you prioritize for your efforts towards diagnostic stewardship today?” by laboratory type of respondent.

| **Response: test category** | **Academic Medical Center Laboratory (n=68)** | **Community Hospital Laboratory (n=28)** | **Hospital Reference Laboratory (n=18)** | **Other/ Multiple Laboratories (n=5)** | **Total (n=119)** |
| --- | --- | --- | --- | --- | --- |
| Syndromic panel | 15 (22.1%) | 5 (17.9%) | 6 (33.3%) | 1 (20.0%) | 27 (22.7%) |
| Sequencing/mNGS | 21 (30.93%) | 2 (7.1%) | 1 (5.6%) | 1 (20.0%) | 25 (21.0%) |
| Culture | 13 (10.1%) | 3 (10.7%) | 7 (38.9%) | 1 (20.0%) | 24 (20.2%) |
| Serology | 5 (7.4%) | 1 (3.6%) | 2 (11.1%) | 0 (0%) | 8 (6.7%) |
| AST | 5 (7.4%) | 2 (7.1%) | 0 (0%) | 0 (0%) | 7 (5.6%) |
| *C. difficile* | 1 (1.5%) | 0 (0%) | 2 (11.1%) | 0 (0%) | 3 (2.5%) |
| Ova & parasite exam | 0 (0%) | 2 (7.1%) | 1 (5.6%) | 0 (0%) | 3 (2.5%) |
| PCR | 0 (0%) | 0 (0%) | 3 (16.7%) | 0 (0%) | 3 (2.5%) |
| Other^a^ | 3 (4.4%) | 1 (3.6%) | 3 (16.7%) | 1 (20.0%) | 8 (6.7%) |
| No response | 5 (7.9%) | 3 (10.7%) | 2 (11.1%) | 1 (20.0%) | 11 (9.2%) |

Abbreviations: AST, antimicrobial susceptibility testing; mNGS, metagenomic next-generation sequencing

^a^ Includes non-microbiology tests (CRP, n=1) and non-specific tests or categories: stool testing (n=2), high cost molecular (n=1), high volume tests (n=1), LIS support (n=1), any send out (n=1), MALDI (n=1)
